# Pedestrian orientation dynamics from high-fidelity measurements

**DOI:** 10.1038/s41598-020-68287-6

**Published:** 2020-07-15

**Authors:** Joris Willems, Alessandro Corbetta, Vlado Menkovski, Federico Toschi

**Affiliations:** 1 Department of Applied Physics, Eindhoven University of Technology, 5600 MB Eindhoven, The Netherlands; 2 Department of Mathematics and Computer Science, Eindhoven University of Technology, 5600 MB Eindhoven, The Netherlands; 3 Department of Applied Physics, Eindhoven University of Technology, 5600 MB Eindhoven, The Netherlands; 4 Department of Mathematics and Computer Science, Eindhoven University of Technology, 5600 MB Eindhoven, The Netherlands; 5 CNR-IAC, 00185 Rome, Italy

**Keywords:** Applied physics, Imaging techniques, Nonlinear phenomena

## Abstract

We investigate in real-life conditions and with very high accuracy the dynamics of body rotation, or yawing, of walking pedestrians—a highly complex task due to the wide variety in shapes, postures and walking gestures. We propose a novel measurement method based on a deep neural architecture that we train on the basis of generic physical properties of the motion of pedestrians. Specifically, we leverage on the strong statistical correlation between individual velocity and body orientation: the velocity direction is typically orthogonal with respect to the shoulder line. We make the reasonable assumption that this approximation, although instantaneously slightly imperfect, is correct on average. This enables us to use velocity data as training labels for a highly-accurate point-estimator of individual orientation, that we can train with no dedicated annotation labor. We discuss the measurement accuracy and show the error scaling, both on synthetic and real-life data: we show that our method is capable of estimating orientation with an error as low as $7.5^\circ$. This tool opens up new possibilities in the studies of human crowd dynamics where orientation is key. By analyzing the dynamics of body rotation in real-life conditions, we show that the instantaneous velocity direction can be described by the combination of orientation and a random delay, where randomness is provided by an Ornstein–Uhlenbeck process centered on an average delay of $100\,\hbox {ms}$. Quantifying these dynamics could have only been possible thanks to a tool as precise as that proposed.

## Introduction

The orientation of our body and shoulder-line changes continuously as we walk. When our gait is regular, these changes are nearly periodic and follow the swinging trend of our trajectories as we balance our weight between our feet^1^. At times, motion direction and body orientation remain temporarily decoupled. This happens, for instance, when we sidestep or in proximity of turns and distractions.

Shoulder-line yawing is not just a mechanical reflection of the walking action, it rather becomes an essential dynamic ingredient as our motion gets geometrically constrained, e.g. by a dense crowd or by a narrow environment. In both cases, as we need to make our way to our destination, we, consciously or unconsciously, rotate our bodies sideways to minimize collisions or maintain comfort distances with other pedestrians or the environment. The capability of measuring and understanding the orientation dynamics of our body and shoulders comes both with societal and fundamental relevance. As a proxy for sight direction, shoulder orientation can be used to assess individual visual attention^2^ or even to increase our capacity to identify anomalous behavior. Moreover, augmenting the traditional position-centered modeling of pedestrians with the orientation degree of freedom, strengthens the connection between human dynamics and other active matter systems, where shape and nematic ordering are key elements to individual and collective behaviors, particularly at high densities (e.g.,^3,4^).

**Figure 1:**
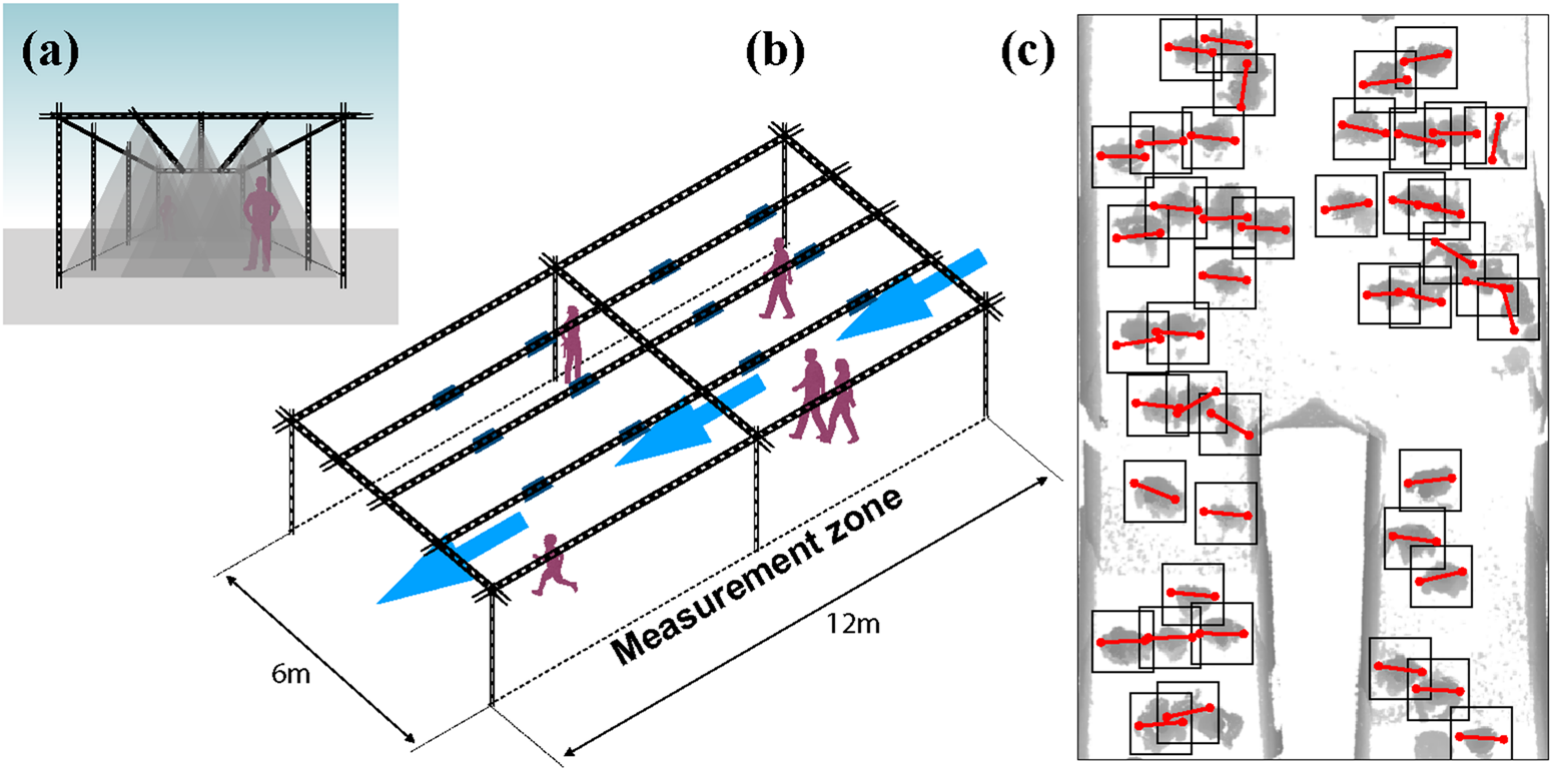
We measure and investigate the dynamics of shoulder orientation for walking pedestrians in real-life scenarios. Our measurements are based on raw data acquired via grids of overhead depth sensors, such as Microsoft Kinect™^5^. In (**a**, **b**) we report, respectively, a front and an aerial view of a data acquisition setup (similar to that in Ref.^6^). The sensors, of which the typical view cone is reported in (**a**), are represented in (**b**) as thick segments. In overhead depth images (**c**), the pixel value, here colorized in gray, represents the distance between each pixel and the camera plane: brighter shades are far from the sensor and, the darker the pixel color, the closer the pixel is to the sensor. Heads are, therefore, in darker shade than the floor. Through localization and tracking algorithms from Refs.^7,8^, we extract imagelets centered on individual pedestrians (cf. imagelets annotated with ground truth in Fig. 2) for which we estimate orientations via the method introduced here.

The dynamics of shoulder-line rotation has been scarcely investigated from a quantitative viewpoint. The data currently available is extremely limited and has been acquired via few laboratory experiments (e.g. Refs.^9–11^). Such scarceness of accurate data hinders the capability of statistic characterizations of the rotation dynamics beyond the estimation of average properties, to include, e.g., fluctuations and rare events. We believe that this is connected with the inherent technical complexity of measuring body yawing accurately and in real-life conditions. Real-life measurements campaigns, in fact, need to rely only on non-intrusive imaging data (or alike) of pedestrians, and cannot be supported by ad hoc wearable sensors, such as accelerometers^10^. Indeed, even the accurate estimation of the position of an individual in real-life, a more “macroscopic” or “coarser-scale” degree of freedom than orientation, is a recognized technical challenge^12^. Since few years, overhead depth-sensing^7,8,13,14^, as used in this work, has been successfully employed to perform accurate pedestrian localization and prolonged tracking campaigns (see example in Fig. 1 and Ref.^6^). Overhead depth data, not only allows privacy respectful data acquisition, but enables also accurate position measurements even in high-density conditions (for a highly-accurate algorithm leveraging on machine learning-based analyses see, e.g. Ref.^15^).

**Figure 2:**
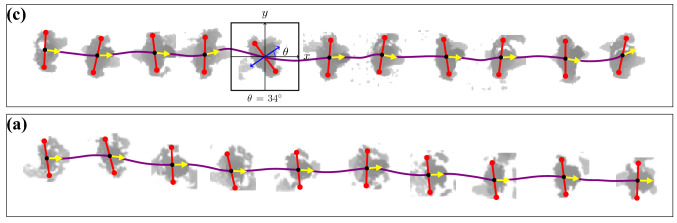
(**a**, **c**) Pedestrian trajectories (purple) superimposed to depth snapshots (gray). Orientation estimates and local velocities (directions of motion) are reported, respectively, in red and yellow. We estimate shoulder orientation on a snapshot-by-snapshot basis, considering depth “imagelets” centered on a pedestrian. The sub-panel (**b**) reports an example of such an imagelet with the $x-y$ coordinate system considered. We employ instantaneous direction of motion $\theta _v$ extracted from preexisting trajectory data as training labels for a neural network. This yields a reliable estimator for the orientation $\theta$, accurate even in cases challenging for humans, like in (**c**). Due to clothing, arms and body posture, presence of bag-packs or errors in depth reconstruction, the overhead pedestrian shape might appear substantially different from an ellipse elongated in the direction of the shoulders.

In this paper we propose a novel method to measure—in real-life conditions and with very high accuracy—the shoulder rotation of walking pedestrians. Our measurement method is based on a deep Convolutional Neural Network^16^ (CNN) point-estimator which operates on overhead depth images centered on individual pedestrians—from now on referred to as “imagelets”. Intuition suggests that pedestrians seen from an overhead perspective have a well-defined elongated, elliptic-like, shape. In our measurements this is true only in a small fraction of cases in which pedestrians walk carrying their arms alongside the body. Conversely, we found a majority of exceptions, impossible to address by hand-made algorithms (cf. Fig. 2). This marks an ideal use-case for supervised deep learning^16^.

It is well known that the high performance of Deep Neural Network methods come also at the price of, often prohibitively, labor-intensive manual annotations of training data (frequently in the order of millions of individual images). Depending on the context, the reliability of human annotations can furthermore be arguable, this is the case whenever different experts are in frequent mutual disagreement about the annotation value. Shoulder orientation in depth imagelets falls in such a case. Here by relying on the strong statistical correlation between individual velocity and body orientation, we manage to produce potentially limitless annotations. While walking on straight paths, our velocity direction is (on average) in very good approximation orthogonal to our shoulder line. On this basis, we can employ the velocity direction as a singularly slightly imperfect, but correct on average, annotation for the orientation. Notably, the zero-average residual error between the velocity direction and the actual orientation gets averaged out as we train our CNN point-estimator with gradient descent. This (self) amends for annotation errors.

We investigate the orientation measurement accuracy of our method and consider its error scaling vs. the size of the training set using both real-life and synthetic depth imagelets. Combining extensive training with the enforcement of *O*(2) symmetry of the estimator, we show that we can deliver an orientation estimator with an error as low as $7.5^\circ$. Our tool enables us to characterize the stochastic process that connects the instantaneous velocity direction to the shoulder orientation. We show that the velocity orientation can be well described by delaying the orientation dynamics through a stochastic process centered on, about, $100\,\hbox {ms}$ and with Ornstein–Uhlenbeck (OU) statistics.

Conceptually speaking, although our tool has been devised for depth imagelets, it can be easily extended to other computer vision-based pedestrian tracking approaches and, more in general, can be used for any system in which there is a statistical connection between (average) individual “particle” velocity and (average) shape.

## Orientation measurements: problem definition

Let $\mathcal {I}$ be a overhead imagelet centered on a pedestrian, see examples in Fig. 2 (for convenience we opt for imagelets of square shape, yet this is not a constraint).

We define the shoulder-line orientation angle, $\theta$, as the angle between the direction normal to the shoulder-line and a fixed reference, here the *y* axis (direction $\mathbf {e}_y$, cf. Fig. 2a,b). According to this definition, a body rotation of $180^\circ$ leaves $\theta$ unchanged. Thus, we aim at a function *f* such that1where $f(\mathcal {I}) = \theta _o$ approximates the actual orientation $\theta$ (with $-\pi /2\equiv \pi /2$, i.e. $\theta$ is an element of the real projective line $\mathbf{P} ^1(\mathbb {R})$, see e.g. Ref.^17^). We report a list of the symbols employed in Table 1.

**Table 1:** List of symbols

Symbol	
$\mathcal {I}$	Overhead imagelet, centered on a pedestrian
*f*	Orientation estimator, modelled via a neural network
$\mathbf{P} ^1(\mathbb {R})$	Real projective line, the set on which we consider pedestrian orientation
$\mathbb {E}[x]$, $\mathbb {E}_y[x]$	Average value of *x* (probability law and/or set are indicated as subscript, *y*, if necessary)
$h_{pred}$	Network estimate of $\theta$ as a discrete probability distribution
$h_2(\theta _v)$	“Two-hot” discrete probability distribution encoding for $\theta _v$ (in training)
$\mathcal {H}(\cdot ,\cdot )$	Cross-entropy loss
$\mathbf {v}(t)$	Instantaneous velocity
$\theta$	Ground-truth shoulder-line orientation angle
$\theta _v$	Velocity direction angle
$\theta _o$	Orientation point estimation, as predicted by the network
$\theta (t), \theta _v(t)$	Low-pass filtered continuous time signals of, respectively, $\theta_0$ and $\theta _v$
$\epsilon$	Symmetric, zero-centered residual that relates $\theta$ and $\theta _v$
*O*(2)	Orthogonal group, containing all rotations and mirrorings that can be applied to $\mathcal {I}$
$\tilde{f}$	Orientation estimator, strictly respecting *O*(2) symmetry
$\hat{b}$	Average prediction bias, quantifying the network systematic error
ARMSE	Average root mean square error, quantifying the total network error
$\theta _{gt}$	Ground-truth orientation annotation, available only for synthetic data
$\theta _r$	Reference annotation for real-life data. Obtained by subsampling from smoothed orientation signals $\theta (t)$
$\hat{v}$	Average walking velocity
*d*(*t*)	Simulated delay $\theta (t)$ and $\theta _v(t)$
*A*	Simulation parameter, relating amplitudes of $\theta (t)$ and $\theta _v(t)$
$\hat{d}$	Simulation parameter, average delay of 80 ms between $\theta (t)$ and $\theta _v(t)$
$\xi$	Simulation parameter, intensity of the $\delta$-correlated noise
$\tau$	Simulation parameter, relaxation time of OU-process
$\dot{W}$	$\delta$-Correlated white noise

We model the mapping *f* via a deep neural network that we train in a supervised, end-to-end, fashion (see structure in the Supporting Information, SI). The network returns the estimate of $\theta$ as a discrete probability distribution, $h_{\mathrm{pred}}$, on $\mathbf{P} ^1(\mathbb {R})$ (quantized in $B=45$ uniform bins, $4^\circ$ wide, via soft-max activation function in the final layer). We retain the $\mathbf{P} ^1(\mathbb {R})$-average (“circular average”) of the distribution $h_{\mathrm{pred}}$, as final output. It formulas, the output $\theta _o$ reads

2 $$\begin{aligned} \theta _o = \mathbb {E}_{\theta '\sim h_{\mathrm{pred}}, \mathbf{P} ^1(\mathbb {R})}[\theta '], \end{aligned}$$

we leave the details to the Supplementary information.

We train with orientation data with a “two-hot” encoding: each orientation $\theta$ is unambiguously represented in terms of a probability distribution non-vanishing on (up to) two adjacent angular bins (we chose “two-hot” in opposition to the typical one-hot training data for classification problems, in which the annotations are Dirac probability distributions on the ground-truth class). We will refer to this encoding, that avoids quantization errors, as $h_2(\theta )$ (we observed no strong sensitivity on the number of bins when these were more or equal than 10). As usual, we use a cross-entropy loss, $\mathcal {H}(\cdot ,\cdot )$.

**Figure 3:**
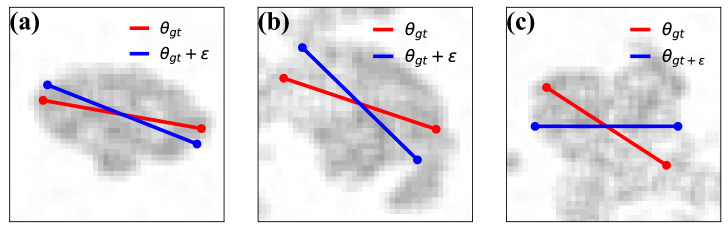
Examples of synthetic imagelets that we employ to analyze the performance of our neural network. Contrarily to the real-life data, ground-truth orientation is available for synthetic data, enabling accurate validation of the estimations. The neural network is trained against labeled target data with predefined noise level ($\sigma = 20^\circ$) to imitate training with real-life imagelets and velocity target data. Target data with predefined noise level and ground-truth orientation for validation are superimposed on the imagelets as blue and red bars respectively.

We employ pedestrian velocity information to tackle the need for huge amounts of accurately annotated data to train the free parameters of the deep neural network (usually in the millions, $\approx 1.3\;{\text {M}}$ in our case).

Let $\theta _v(t) \in \mathbf{P} ^1(\mathbb {R})$ be the angle between the walking velocity and a reference at time $t>0$, i.e.

3 $$\begin{aligned} \theta _v(t) = \angle (\mathbf {v}(t),\mathbf {e}_y), \end{aligned}$$

where $\mathbf {v}(t)$ is the instantaneous velocity, and $\angle (\cdot ,\cdot )$ denotes the angle comprised the directions in its argument (with $\pi$-periodicity). When we walk, either for the periodic sway or when we make turns, our shoulder line is most-frequently, and in very good approximation, orthogonal with respect to the walking velocity, i.e.

4 $$\begin{aligned} \theta \approx \theta _v. \end{aligned}$$

Therefore, velocities provide a meaningful “proxy” annotation for orientation. We used the “approximately equal” sign in Eq. (4) because we can have frequent, yet small, disagreements between velocity and orientation. These can be due to small loss of alignment between the two (e.g. because something attracted our attention) or they can be due to inaccuracies, e.g., in the velocity measurements. It is also possible, yet less likely, that velocity and orientation remain misaligned for longer time intervals. This holds, e.g., for people walking sideways. We retain these as rare occasions, which we expect to occur symmetrically for both left and right sides, with no relevant weight in our training dataset. This hypothesis reasonably holds on unidirectional pedestrian flows happening on rectilinear corridors, but might be invalid in case, e.g., of curved paths. Formally, for a walking person, we model the relation in Eq. (4) as

5 $$\begin{aligned} \theta = \theta _v + \epsilon , \end{aligned}$$

with $\epsilon$ being a small, symmetric, and zero-centered residual.

We train our neural network using the labels $h_2(\theta _v)$ as a proxy for $h_2(\theta )$. The training process aims at the minimization of the (average) loss $\mathbb {E}_{\theta _v}[\mathcal {H}(h_{pred},h_2(\theta _v))]$. As such, the output $h_{pred}$ converges to the distribution of annotations of similar imagelets, whose average is the correct point-estimation of the orientation:

6 $$\begin{aligned} \theta _o \approx \mathbb {E}_{\theta ' \sim \theta _v + \epsilon , \mathbf{P} ^1(\mathbb {R})}[\theta '] = \mathbb {E}[\theta - \epsilon ] = \theta . \end{aligned}$$

We refer to the Supplementary information for a formal proof with simplifying assumptions and a simulation-based proof in the general case.

Finally, once a pedestrian with shoulder orientation $\theta$ rigidly rotates around the vertical axis by an angle $\alpha$, their orientation becomes $\theta + \alpha$. Similarly, “mirroring” a pedestrian around the $\mathbf {e}_y$ direction, their orientation changes sign.

The map *f* must satisfy such symmetry with respect to imagelets rotations and mirroring. In other words, *f* must be co-variant^18^ with respect to the group, *O*(2), of the orthogonal transformations of the plane. In formulas, this reads

7 $$\begin{aligned} f(\phi \mathcal {I}) = (f(\mathcal {I}) + \alpha )\det (\phi ) \end{aligned}$$

for all transformations $\phi =\Phi R_\alpha \in O(2)$, that concatenate a rotation of $\alpha$, $R_\alpha$, and, possibly, a reflection (i.e. $\Phi \in \{\text {Id}, J\}$, respectively the identity, $\text {Id}$, and the reflection, *J*, from which the sign change given by the determinant of the transformation: $\det (\phi )=\det (\Phi )=\pm 1$).

Symmetries in neural networks are often injected at training time, by augmenting the training set by all the symmetry group orbits. Similarly, we include multiple copies of the same imagelets with multiple random rotations with and without flipping. This also ensures that the training set spans $\mathbf{P} ^1(\mathbb {R})$ uniformly. Yet, this does not yield a strictly *O*(2)-symmetric estimator Eq. (7). We further enforce this symmetry by constructing a new map, $\tilde{f}$, as the *O*(2)-group average of *f*, which is thus strictly respecting Eq. (7). In formulas it holds

8 $$\begin{aligned} \tilde{f}(\mathcal {I})&= \frac{1}{|O(2)|}\int _{O(2)}(f(\phi \mathcal {I}) - \alpha )\det (\phi )\, d\phi , \end{aligned}$$

9 $$\begin{aligned}&=\frac{1}{2\pi }\int _{0}^{2\pi }\frac{f(R_\alpha \mathcal {I}) - f(JR_\alpha \mathcal {I})}{2} - \alpha \, d\alpha , \end{aligned}$$

we leave the proof of this identity, the *O*(2)-symmetry of $\tilde{f}$ and further details on $\mathbf{P} ^1(\mathbb {R})$-averages to the SI. In the following, we consider approximations of the integral in Eq. (9) by equi-spaced and random sampling of *O*(2).

**Fig. 4:**
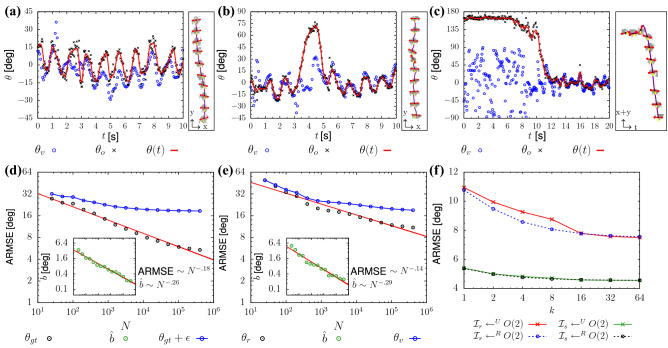
(**a**–**c**) Velocity direction and shoulder orientation signal, for three trajectories collected in real-life (depth maps sequences similar to Fig. 2 are on the right of each panel). We report the instantaneous values of velocity (obtained from tracking) and predicted orientation, $\theta _v$ and $\theta _o$, and the continuous orientation signal $\theta (t)$ (low-pass filter of $\theta _o$). Orientation has been computed via our CNN trained on 30 h of real-life velocity data. (**a**) Reports a typical pedestrian behavior, where $\theta _v(t)$ and $\theta (t)$ oscillate “in sync” (frequency $f \approx$ $0.8\,\hbox {Hz}$) following the stepping. Our tool resolves correctly also rare sidestepping events or orientation of standing individuals in which the signals are out of sync. (**b**) Shows a pedestrian rotating their body, possibly observing their surroundings, while maintaining the walking direction. (**c**) Shows an individual initially standing, then performing a $150^{\circ }$ body rotation and finally walking away. In this case, the velocity $\theta _v$ is undefined for time $t<10\,\hbox {s}$ as there is no position variation (so high noise intensity $\theta _v$). Note that the depth map sequences in (**c**) are in space-time coordinates, in a photo finish-like fashion. In this reference, when the pedestrian stands still, a horizontal $y=const$ line is traced. (**d**–**f**) Prediction performance of the network, *f* (Eq. 1), in case of artificial imagelets (**d**) and real-life data (**e**). We train with datasets of increasing size (*N*, *x*-axis). We report the Root Mean Square Error of the predictions averaged over $M=32$ independent training of the networks [ARMSE, Eq. (11)] and, in the inset, the average bias, $\hat{b}$ (Eq. 10). The test sets used to compute the indicators include, for (**d**), 25k unseen synthetic images with error-free annotation ($\theta _{gt}$) and, for (**e**), 25k unseen real-life imagelets, annotated considering low-pass filtered high-resolution orientation estimates, $\theta _r$, obtained with our neural network trained with $1\,\hbox {M}$ samples and *O*(2)-group averaging. The bias, in both cases, decreases rapidly below $0.1^\circ$. The ARMSE, for the networks trained with the largest dataset approaches, respectively, $5.5^\circ$ and $11^\circ$, as *N* grows. For both ARMSE and bias, we report the fitted exponents characterizing the error converge in the label. We complement the evaluation of the ARMSE considering noisy labels [$\theta _{gt} +\epsilon$ for case (**d**) and $\theta _v$ for case (**e**)]. In case (**d**) the ARMSE saturates consistently with the level of noise in the labels (cf. SI). In case (**e**), the ARMSE approaches a saturation point at about $20^\circ$. This reflects the random disagreement between velocity and orientation. (**f**) Performance can be further increased by enforcing *O*(2)-symmetry of the orientation estimator, map $\tilde{f}$, Eq. (8). In (**f**) we consider maps $\tilde{f}$ built from the networks trained with the largest training datasets from (**d**, **e**) ($N\approx 0.5\,\hbox {M}$), both for the synthetic and real-life cases, vs. the number of samples used for the group average, *k*. We consider both uniform and random sampling of *O*(2) (superscript *U* and *R* respectively). The group average further reduces the ARMSE from $5.5^\circ$ to $4.5^\circ$ in case of synthetic imagelets $\mathcal {I}_s$ (no observable difference between uniform and random sampling), and from $11^\circ$ to $7.5^\circ$ in case of real-life imagelets $\mathcal {I}_r$, with higher performance in case of random sampling for $k < 16$.

**Fig. 5:**
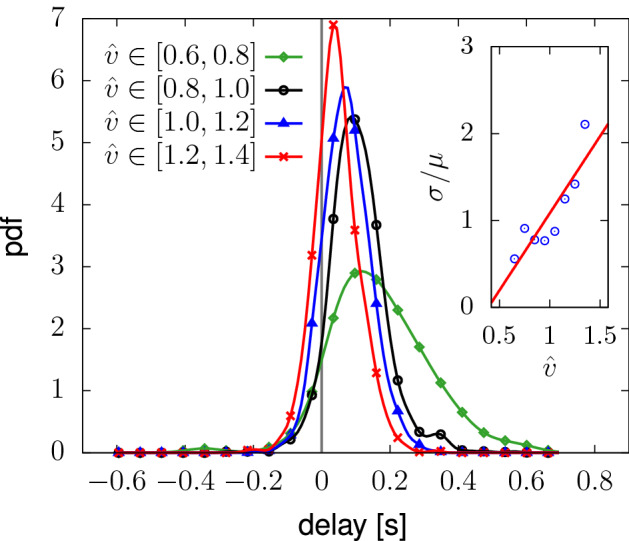
Probability distribution function of the delay time between the shoulder orientation, $\theta (t)$, and velocity orientation, $\theta _v(t)$, signals for different average velocities, $\hat{v}$. As the average velocity grows, the average delay and the delay fluctuations reduce. The inset reports the ratio between the standard deviation, $\sigma$, and the average, $\mu$, of the delay as a function of $\hat{v}$. The measurements considered are 78k trajectories ($20\,\hbox {h}$ of data, all not exceeding a maximum orientation of $\pm 60^\circ$), acquired during the GLOW event^6^.

## CNN: training and testing

We consider two types of training/testing imagelets: algorithmically generated, “synthetic”, imagelets, of which the orientation angle $\theta$ is known, and real-life imagelets. In the first case we mimic a velocity-based training by adding a centered noise to labels known exactly [following Eq. (5)]. In the second case, as we have no manually annotated ground truth, of which the accuracy would nevertheless be debatable, we propose a validation based on the convergence towards low-pass filtered orientation signals. In both cases, we show that the average prediction error [ARMSE, Eq. (11)] is about $7.5^\circ$ degrees or, possibly, lower, should the training set size *N* be large enough. Specifically, the datasets are as follows:

### Synthetic dataset

We generate synthetic imagelets mimicking the overhead shape of people in terms of a superposition of two ellipses: one for the body/shoulders, $E_b$, and another one, $E_h$, at lower depth values (i.e. higher on the ground), for the head. We generate synthetic imagelets mimicking the overhead shape of people in terms of a superposition of two ellipses: one for the body/shoulders, $E_b$, and another one, $E_h$ at lower depth values (i.e. objects higher above the ground are closer to the overhead mounted depth sensors and are thus associated with smaller depth values), for the head.

We report examples of such imagelets in Fig. 3, while the details of the generation algorithm are left to the SI.

By construction, the rotation angle $\alpha _{b}$ of $E_b$ represents the pedestrian orientation, i.e. it is the ground truth for the training. We train the network with such synthetic imagelets and a small centered Gaussian noise $\epsilon \sim \mathcal {N}\left( 0^\circ , 20^\circ \right)$ superimposed to $\alpha _{b} = \theta _{gt}$ to imitate velocity-based training. Hence, we train using labels $\alpha _{gt} + \epsilon$ while we validate with $\alpha _{gt}$ (cf. Eq. 5).

### Real-life dataset

We consider depth images and velocity data from a real-life measurement campaign conducted during a city-wide festival (GLOW) in Eindhoven, The Netherlands, in Nov. 2017. The measurements involve a uni-directional crowd flow passing through a corridor-shaped exhibit (tracking area: $12\;{\text {m}} \times 6\;{\text {m}}$), for further details see^6^. The dataset leverages on high-resolution individual localization and tracking based on overhead depth images (as in Fig. 1) and with $30\,\hbox {Hz}$ time sampling. The localization and tracking algorithms employed are analogous to what employed in previous works^7, 8^. To ensure that our velocity data provides a well-defined proxy for orientation, we restrict to pedestrians having average velocity above $0.65\,\hbox {m/s}$. Moreover, for each trajectory we extract imagelets and velocity data with a time sampling of $\Delta T \approx 0.5\,\hbox {s}$, which increases the independence between training data.

Additionally, we apply random rotations and random horizontal flips to all imagelets (and, correspondingly, to labels). This aims at training with a dataset uniformly distributed on $\mathbf{P} (\mathbb {R}^1)$.

In absence of ground truth, we build our test set as follows: we rely on our neural network trained with $1\,\hbox {M}$ different imagelets (i.e. twice as much the largest training dataset considered in Fig. 4d,e, on which we perform random augmentation and final *O*(2)-averaging of the operator), hence the most accurate, to make orientation predictions over complete pedestrian trajectories. As an orientation signal $\theta (t)$ needs to be continuous in time, we smoothen the predicted $\theta (t)$ in time (low-pass Butterworth filter^19^ of order $n=1$, cutoff frequency $c_f=2.0\,\hbox {Hz}$ and window length $l=52$) to eliminate random noise. The final dataset contains values $\theta (t)$ from different trajectories and sampled at different, independent, time instants. Therefore, we build the dataset on the basis of two independent elements: a heavily trained network and a physics-based time-regularity hypothesis on orientation signals.

We assess the prediction performance as the training set size, *N*, increases. To compute exhaustive performance statistics, we train the network on *M* independent datasets for every *N* (in a cross-validation-like setting). We can distinguish two kinds of errors, systematic and random^20^. The first is an error that always, and in the same manner, interferes with the outcome of the measurements (e.g. a constant rotation offset for all predictions); the second, also referred to as variance, is caused by unexplained variability of the model with respect to the observed imagelets (i.e. the prediction accuracy may vary between different imagelets). To quantify the network performance in relation to these two sources of error, we employ the two following measures. Given a reference orientation (e.g. ground truth), $\theta _r(\mathcal {I})$, for an imagelet $\mathcal {I}$ from dataset $D_k$ ($k=1,2,\dots ,M$), we quantify the systematic error as the average prediction bias, $\hat{b}$, evaluated as the root-mean-square of the individual network biases, $\hat{b}_k$:

10 $$\begin{aligned} \hat{b} = \sqrt{\frac{1}{M}\sum _{k=1}^M{ \hat{b}^2_k }} = \sqrt{\frac{1}{M}\sum _{k=1}^M{ \left( \mathbb {E}_{\mathcal {I}\in D_k}[\theta _o(\mathcal {I}) - \theta _r(\mathcal {I})]\right) ^2 }}. \end{aligned}$$

Additionally, we consider the average root-mean-square error (ARMSE), which quantifies the total error, as superposition of systematic and random components. In formulas, this reads

11 $$\begin{aligned} \text {ARMSE} = \frac{1}{M} \sum _{k=1}^M\sqrt{\hat{b}^2_k + V_k} = \frac{1}{M} \sum _{k=1}^M \sqrt{\mathbb {E}_{\mathcal {I}\in D_k}[ (\theta _o(\mathcal {I}) - \theta _r(\mathcal {I}))^2 ]}, \end{aligned}$$

where $V_k$ is the variance of the prediction of the *k*-th network.

**Fig. 6:**
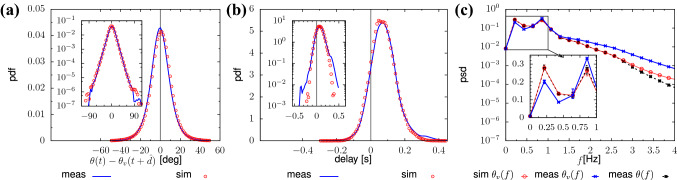
Comparison between simulations (red dots) and real-life measurements (blue solid line). We build velocity direction signals $\theta _v(t)$ on top of delayed orientation measurements $\theta (t-d(t))$, where the delay *d*(*t*) is modelled by a OU random process [cf. Eqs. (12), (13)]. Measurements have been acquired during the GLOW event (36k trajectories, restricting to people keeping normal average velocity $\hat{v} \approx 1.3\,\hbox {m/s}$). In (**a**) we report the probability distribution function (pdf) of the difference between velocity direction and orientation shifted in time by the average delay, $\hat{d} = 0.1\,\hbox {s}$. In (**b**), analogously to Fig. 5, we report a PDF of the delay time between $\theta (t)$ and $\theta _v(t)$. The insets in (**a**, **b**) report the data in semi-logarithmic scale. For both these quantities we observe excellent agreement among simulations and measurements for (**a**, **b**). Panel (**c**) shows the velocity direction signals’ grand average power spectral density (psd) of $\theta _v(t)$ and $\theta (t)$. Our model modifies the psd only at high frequencies. As an effect, the most energetic components of the velocity orientation, around $0.2\,\hbox {Hz}$ and $1\,\hbox {Hz}$, remain, respectively slightly under and slightly over-represented.

## Results

In Fig. 4a–c, we report the orientation signals as estimated by the networks in three different real-life contexts. The network is capable of accurate predictions that, as expected, are independent of the actual instantaneous velocity. Hence, it remains accurate in case of a pedestrian walking sideways (Fig. 4b), in which the orientation signal loses temporarily coupling with the velocity orientation and in case of a pedestrian temporarily stopping and standing (Fig. 4c), in which the velocity orientation is even undefined (note that these cases were excluded from the training).

We include in Fig. 4d–f the values of average prediction bias and ARMSE as the training set size increases, in case of synthetic and real-life imagelets [respectively, in (**d**) and (**e**)]. In both cases the network performance increases with *N*, with slightly slower convergence rate for the ARMSE for the real-life dataset, which is likely more challenging to learn than the synthetic one. In both cases the predictions are free of bias (cf. sub-panels). With the largest number of training imagelets considered ($N\approx 5 \times 10^5$), we measured an ARMSE of about $5^\circ$ for the synthetic data and $11^\circ$ for the real-life data. We managed to further reduce this error to, respectively, $4.5^\circ$ and $7.5^\circ$ by enforcing *O*(2) symmetry. Note that we could trivially apply Eq. (8) as we are in a bias-free context, else a systematic correction for the bias would have been necessary. In Fig. 4f, we report the network performance as we approximate better and better the *O*(2) group average. We stress that in case of real-life data, the network predictions, on which no time-smoothing has been applied, converge to test data that underwent time-smoothing. Thus, as *N* grows, the network predictions show increasing robustness and consistency approaching jitter-free signals.

## Real-life orientation dynamics

We are now capable of investigating with high-resolution, and in real-life conditions, the connection between shoulder orientation and velocity direction—which, in the previous sections, we reduced to the error term $\epsilon$. In particular, we can characterize a stochastic delay signal, *d*(*t*), which allows us to model the relation between velocity and orientation as

12 $$\begin{aligned} \theta _v(t) = A\, \theta (t-d(t)), \end{aligned}$$

where *A* is a positive constant.

First, thanks to the high-accuracy of our tool, we measure a velocity-dependent delay between velocity orientation and shoulder orientation whose probability distribution function is in Fig. 5 (see Supplementary information for details on the delay measurement algorithm). The velocity orientation follows in time the shoulder yawing, with a delay that decreases (on average) between $160\,\hbox {ms}$ and $100\,\hbox {ms}$ as the average walking velocity, $\hat{v}$, increases from $0.6\,\hbox {m/s}$ to $1.4\,\hbox {m/s}$ (respectively walking speed values in leisure and normal walking regimes, see, e.g. Ref.^21^).

The structure of *d*(*t*) appears well-modeled by a OU process:

13 $$\begin{aligned} \dot{d}(t) = -\frac{\hat{d} - d(t)}{\tau } + \xi \dot{W}, \end{aligned}$$

where $\hat{d}>0$ is the average delay ($\hat{d} \approx \langle d(t) \rangle$), $\tau >0$ is the OU time-scale and $\xi >0$ is the intensity of the $\delta$-correlated white noise $\dot{W}$. In particular, in Fig. 6 we compare statistical observables of measurements and simulations considering the case of normal walking speed (average velocity $\hat{v} \approx 1.3\,\hbox {m/s}$), of which we retain the measured orientation signals, $\theta (t)$, as a basis for Eq. (12) (simulation parameters: $A=1.85$, $\hat{d} =0.08\,\hbox {s}$, $\tau =1.2\,\hbox {s}$ and $\xi =1.85$). In Fig. 6a, we report the pdf of the difference between orientation and velocity orientation when one is shifted in time by, $\hat{d}$, to compensate for the average delay. Measurements and simulations, in excellent mutual agreement, follow a Gaussian statistics. Thanks to a stochastic delay, we achieve a very good quantitative agreement in the delay distributions (Fig. 6b). In Fig. 6c, we report the Power Spectral Density (psd) of $\theta _v(t)$ and $\theta (t)$ computed by averaging all the psds obtained from individual velocity direction and orientation signals. We observe that the stochastic delay does not substantially modify the psd of orientations, especially at low frequencies. As an effect, the peak around $f=1\,\hbox {Hz}$, connected with the periodic swinging in walking (see Fig. 4a), is reproduced (yet it is slightly underestimated). Moreover, the psd shows another peak around $0.2\,\hbox {Hz}$, which is connected to large-scale motion (a pedestrian might curve along their path) and/or have a non-periodic orientation signal (which yields low-frequency spectral artifacts). Also this peak, not modeled by Eq. (13), is reproduced by the model but with a slight overestimation.

## Discussion

In this paper we presented an extremely accurate estimator for the pedestrian shoulder-line orientation based on deep convolutional neural networks. We leveraged on statistic aspects of pedestrian dynamics to overcome two outstanding issues related to deep networks training: the labor-intensive annotation of training data in sufficient amounts (generally millions of images) and the accuracy of annotations in non-trivial contexts.

Thanks to the strong statistical correlation of shoulder-line and velocity direction, which are typically orthogonal, we can employ the velocity direction as a training label. Although often slightly incorrect, it remains correct on the average, to which our point-estimator converges. Notably, the relation between velocity and orientation holds regardless of the quality of the raw imaging data employed. In case of overhead depth maps, as used here, often we had disagreement between human annotators, which would unavoidably yield low quality labels. By using velocity we can circumvent this issue and produce training data in arbitrarily large amounts. We stress that this correlation assumption is crucial only for training the estimator. As evidenced in the paper, once trained, the estimator can be used to successfully measure pedestrian orientation when shoulder-line orientation and velocity direction are systematically not orthogonal (like it happens for people walking sideways), or even for vanishing walking velocity (where the velocity orientation is not defined). We mention additionally that our approach can be conceptually extended to other imaging formats, such as color images, provided accurate and sufficiently prolonged tracking data are available.

Our tool unlocked the possibility to accurately investigate the relation between velocity direction and shoulder orientation. We could measure a velocity-dependent delay of about $100\,\hbox {ms}$ between velocity and orientation, that we are able to quantitatively reproduce in terms of a simple Ornstein–Uhlenbeck process. In particular, on the basis of measured orientation signals, we could generate velocity directions featuring amplitude with respect to the orientation signal, velocity-orientation delay distribution and power spectral density in very good agreement with the measurements.

We currently employed our velocity-trained network to investigate dynamics at relatively low density. We expect the network to be capable to operate and deliver accurate orientation estimates in different scenarios from those considered. As such, natural next steps are the investigation of static and dynamic high-density crowds, clogged bottlenecks conditions, or other scenarios in which the “nematic” ordering of the crowd is expected to play a key role in the dynamics. Additionally, the tool developed can be applied to do (real-time) analyses of orientation, e.g. to gather a first estimate of sight/attention direction and/or possibly extend anomaly detection capabilities for crowd motion.

## Supplementary information

Supplementary information. (PDF 428 kb)
