# Supplementary material for: Pedestrian orientation dynamics from high-fidelity measurements
Source: Sci Rep. 2020 Jul 15;10:11653. doi: 10.1038/s41598-020-68287-6 (PMC7363920; doi:10.1038/s41598-020-68287-6)
Supplement: Supplementary file 1 — Supplementary information. (PDF 428 kb) [file 41598_2020_68287_MOESM1_ESM.pdf]

Supporting Information
Pedestrian orientation dynamics from high-fidelity measurements

J. Willems[a], A. Corbetta[a], V. Menkovski[b] and F. Toschi[c]

[a]Department of Applied Physics, Eindhoven University of Technology,
5600 MB Eindhoven, The Netherlands
[b]Department of Mathematics and Computer Science, Eindhoven University of Technology,
5600 MB Eindhoven, The Netherlands
[c]Department of Applied Physics, Department of Mathematics and Computer Science,
Eindhoven University of Technology, 5600 MB Eindhoven,
The Netherlands and CNR-IAC, I-00185 Rome, Italy

Arithmetics of angles on $\mathbf{P}^1(\mathbb{R})$

We choose to parametrize the projective line, $\mathbf{P}^1(\mathbb{R})$, with the interval $[-\frac{\pi}{2}, \frac{\pi}{2})$. An angular value, $\theta' \in \mathbb{R}$, is reported to this parametrization of $\mathbf{P}^1(\mathbb{R})$ through the wrap function, defined as

$$\text{wrap}\,(\theta') = \text{mod}(\theta' + \pi/2, \pi) - \pi/2. \tag{S1}$$

We define arithmetic operations such as angle summation and subtraction of two angles, say $\theta_1, \theta_2 \in \mathbf{P}^1(\mathbb{R})$, via Eq. (S1) as

$$\theta_1 \pm \theta_2 = \text{wrap}\,(\theta_1 \pm \theta_2). \tag{S2}$$

Weighted averaging operations on $\mathbf{P}^1(\mathbb{R})$ (e.g. Eq. (2) and Eq. (8)) are computed in this parametrization as

$$\mathbb{E}_{\theta' \sim h(\theta), \mathbf{P}^1(\mathbb{R})}[\theta'] = \frac{1}{2}\,\text{arctan2}\left(\int_{-\pi/2}^{\pi/2} h(\theta)\sin(2\theta)d\theta, \int_{-\pi/2}^{\pi/2} h(\theta)\cos(2\theta)d\theta\right), \tag{S3}$$

where $h(\theta)$ is a probability density function on $\mathbf{P}^1(\mathbb{R})$. Namely, angles θ are converted to corresponding Cartesian points on the unit circle (i.e. $\theta \to (\cos(2\theta), \sin(2\theta))$), then a vector average weighted by $h(\theta)$ is performed, and the final result is mapped back to $\mathbf{P}^1(\mathbb{R})$ via $\frac{1}{2}\,\text{arctan2}(\cdot)$. Note that Eq. (S3) is not defined whenever the vector average vanishes. This happens, for instance, when $h(\theta)$ is uniform. For further details on arithmetic of periodic variables, we refer to [1].

"Two-hot" encoding

Our neural network outputs a discrete probability distribution over $B = 45$ classes. We interpret it as a probability over $[-\pi/2, \pi/2)$ once partitioned in B equal adjacent intervals of size π/B and centered around the mid-value $\theta_i = \pi(1/2B - 1) + i\,\pi/B = -88^\circ + i\,4^\circ$, with $i \in \{0, 1, 2, ..., B-1\}$. Let $h(\theta_i)$ be the considered probability distribution. In support of the required circular properties, we enforce adjacency of the outer bins, such that they "wrap" around $\mathbf{P}^1(\mathbb{R})$, and $h(\theta) = h(\theta + k\pi)$ for all $k \in \mathbb{Z}$ holds.

In the continuous case, $B \to \infty$, a ground truth annotation (or the ideal output of the neural network) is a Dirac delta probability distribution, $\delta(x - \theta)$, centered on the true angle θ. From this, θ can be recovered via the expectation in Eq. (S3): $\theta = \mathbb{E}_{\theta \sim \delta(x-\theta)}[\theta']$. For finite B, we prevent quantization errors by unambiguously encoding angles in (up to) two adjacent bins, from which the name "two-hot" encoding (vs. the "one-hot" encoding in standard classification problems). In particular we encode the angle θ as

$$h_2(\theta)[\theta_i] = \mathcal{N}\begin{cases} 1 - \frac{1}{d}|\theta - \theta_i| & \text{if } |\theta - \theta_i| \leq \frac{\pi}{B} \\ 0 & \text{otherwise} \end{cases} \tag{S4}$$

with $\mathcal{N}$ being a normalization constant and $\theta - \theta_i$ being the wrapped distance (Eq. (S2)) of θ and the mid-angle of the i-th bin. Note that the wrapped difference ensures that both $\theta = -\pi/2$ and $\theta = \pi/2$ are encoded into the same distributions, i.e. the network output remains unchanged for a 180° body rotation. By applying Eq. (S3), we recover the annotation angle θ.

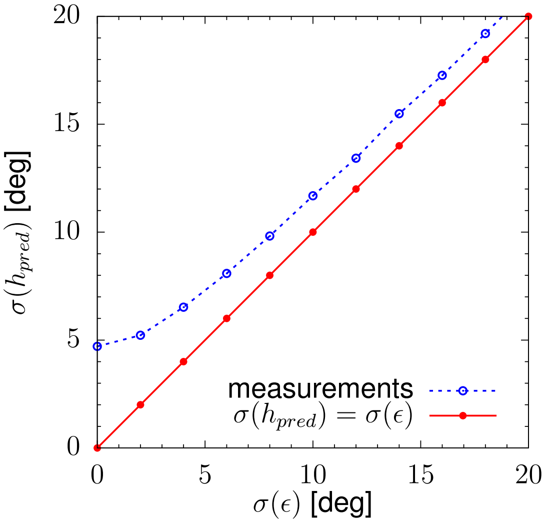

Figure S1: Dispersion (standard deviation) in the neural network output probability distribution $\sigma(h_{pred})$ versus the amplitude (standard deviation) of the error term $\sigma(\epsilon)$ (between training annotations and ground-truth orientation in accordance with Eq. (5)) for synthetic imagelets. We train $M = 16$ networks for each $\sigma(\epsilon) \in \{0, 2, 4, ..., 20\}$ and evaluate $\sigma(h_{pred})$ by averaging the standard deviations of the outputs h_{pred} among the M networks on $10k$ synthetic test imagelets. These measurements are reported as blue dotted lines. We observe good scaling agreement between $\sigma(h_{pred})$ and the theoretical lower limit $\sigma(\epsilon)$ (cf. proof in the text for the simplified case), indicating that our training converges to the average annotation for similar imagelets.

Variance of $h(\theta)$ and cross-entropy based error averaging

In this section we provide a physical meaning to the to the probability distribution predicted by the neural network h_{pred}, beyond the average value, which we retain as predicted angle θ_o. In particular, the standard deviation, $\sigma(h_{\text{pred}})$, scales with the error ϵ of the annotations given to similar imagelets. We prove this claim formally in a simplified case and via simulations in the general one.

Training by minimizing the average cross-entropy, $\mathbb{E}_{\theta_v}[\mathcal{H}(h_{\text{pred}}, \delta_{\theta_v})]$, in the simplified scenario with the following characteristics: 1. $\mathbf{P}^1(\mathbb{R})$ is considered in its discretized version, i.e. $B < \infty$; 2. there is a single training imagelet, $\mathcal{I}$, yet endowed with different (conflicting) annotations; 3. annotations, N in total, are distributed as $\theta_v = \theta + \epsilon$ (where ϵ is zero-averaged, see main text); 4. annotations are centered in some bin $j \in \{0, 1, \ldots, B-1\}$ (i.e. they are Dirac masses, δ_{θ_j}) yields

$$h_{\text{pred}} = \hat{h}_{\theta+\epsilon}, \tag{S5}$$

where $\hat{h}_{\theta+\epsilon}$ is the (discretized) probability distribution of the training data. Notice that, by construction, the $\mathbf{P}^1(\mathbb{R})$-average of h_{pred} is exactly θ.

Proof. Let h_{pred} have values $h(\theta_0), h(\theta_1), \ldots, h(\theta_{B-1})$ on the B bins. The average loss, $\mathcal{L}$, reads

$$\mathcal{L} = \frac{1}{N}\sum_{j=1}^{N}\mathcal{H}(\delta_{\theta_j}, h_{pred}) \tag{S6}$$

$$= -\frac{1}{N}\sum_{j=1}^{N}\sum_{i=0}^{B-1}\delta_{\theta_j}\log h(\theta_i) \tag{S7}$$

$$= -\frac{1}{N}\sum_{j=1}^{N}\log h(\theta_\mu), \tag{S8}$$

where the last equality follows from the definition of Dirac mass.

We can sort and aggregate the elements in the sum in Eq. (S8). In particular, let $\#_j$ be the total number of annotations having value δ_{θ_j}, Eq. (S8) yields:

$$\mathcal{L} = -\sum_j \frac{\#_j}{N} \log h(\theta_j), \tag{S9}$$

by Gibbs' inequality (see, e.g., [2]), it holds

$$-\sum_j \frac{\#_j}{N} \log h(\theta_j) \geq -\sum_j \frac{\#_j}{N} \log \frac{\#_j}{N}. \tag{S10}$$

Therefore, at the absolute minimum for $\mathcal{L}$, h_{pred} is the distribution of the labels, which in our case is $\hat{h}_{\theta+\epsilon}$, i.e.

$$h_{pred} = \sum_j \frac{\#_j}{N} \delta_{\theta_j} = \hat{h}_{\theta+\epsilon} \tag{S11}$$

□

Note that for a two-hot encoding the proof is identical, but each annotation is a convex combination of two delta masses.

In general, in a sufficiently ample dataset of depth imagelets (and related velocity annotation) acquired in absence of biases compromising the relation Eq. (5), we expect to find a wide number of similar imagelets, yet with different annotations, and this provides a rich sampling of the ϵ distribution. Abstracting from the previous proof, we expect the training process to be such that for each set of similar imagelets, the network would learn and output the probability distribution of annotations. We prove this experimentally, by means of synthetic imagelets. In Fig. S1 we compare the amplitude of the symmetric centered error, $\sigma(\epsilon)$, with the standard deviation of the predicted distribution (averaging over a test set of $10,000$ images), showing that they perfectly correlate for $\sigma(\epsilon) > 7°$.

Neural network structure and training

We consider a neural network inspired by the VGG model, whose full structure is in Figure S2. We implemented the network using the Keras library.

We train the network by randomly augmenting the training images at the beginning of every epoch. Specifically, we apply random rotations and random horizontal flips (and we act correspondingly to the associated labels) to all imagelets. This ensures a training dataset uniformly distributed on $\mathbf{P}(\mathbb{R}^1)$. A pre-processing standardization step of the depth intensity is applied individually to all the imagelets.

We employ the Adam optimizer with a batch size of 64, we retain the model that scores the lowest RMSE over a total number of 25 training epochs.

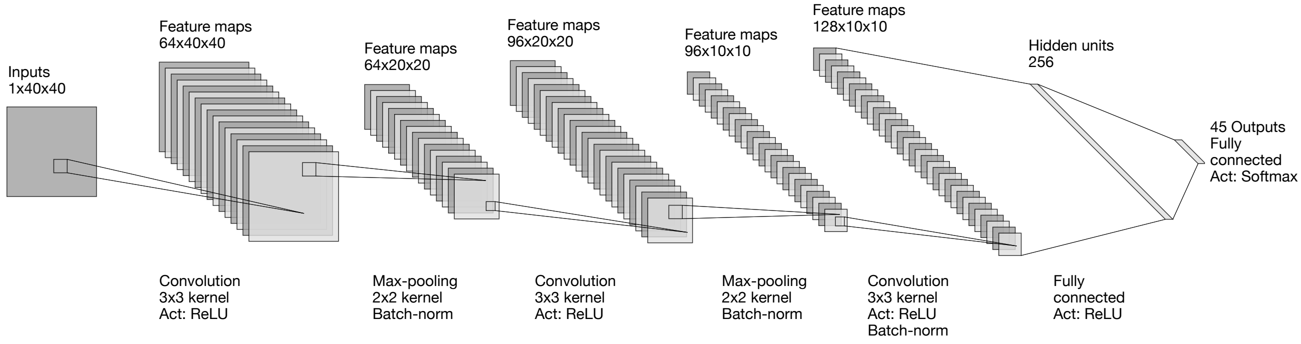

Figure S2: Detailed structure of the neural network. The network is fed with single channel imagelets (40×40 pixels) after which the input data propagates trough two stacks of convolution, max-pooling and batch normalization layers for feature extraction. A convolution and batch-normalization layer connects the feature maps with a fully connected layer (ReLU activation function). The final softmax activation yields a probability mass function $h(\theta)$ on 45 adjacent equal bins as output of the last layer. The network is trained using cross-entropy as loss function.

Map $\tilde{f}$ and $O(2)$-group averaging

In this Section, we deduce identity Eq. (8), and prove that the map $\tilde{f}$ is strictly respecting $O(2)$ symmetry (cf. Eq. (7)).

The identity between Eq. (8) and Eq. (9) can be proved by substitution, considering the fact that $O(2)$ can be decomposed into rigid rotations and rigid rotations applied after a reflection, from which Eq. (S13):

$$\tilde{f}(\mathcal{I}) = \frac{1}{|O(2)|}\int_{O(2)} (f(\phi\mathcal{I}) - \alpha)\det(\phi)\,d\phi \tag{S12}$$

$$= \frac{1}{2}\left(\underbrace{\frac{1}{2\pi}\int_0^{2\pi} f(R_\alpha\mathcal{I}) - \alpha d\alpha}_{\text{rotations: }\det(\phi)=1} + \underbrace{\frac{1}{2\pi}\int_0^{2\pi} -f(JR_\alpha\mathcal{I}) - \alpha d\alpha}_{\text{reflection and rotations: }\det(\phi)=-1}\right) \tag{S13}$$

$$= \frac{1}{2\pi}\int_0^{2\pi}\frac{1}{2}\Big(\big\{f(R_\alpha\mathcal{I}) - f(JR_\alpha\mathcal{I})\big\} - 2\alpha\Big)\,d\alpha \tag{S14}$$

$$= \frac{1}{2\pi}\int_0^{2\pi}\frac{f(R_\alpha\mathcal{I}) - f(JR_\alpha\mathcal{I})}{2} - \alpha\,d\alpha \tag{S15}$$

□

We show here that $\tilde{f}$ respects $O(2)$ symmetry (Eq. (7)).
Proof. Let $\phi\mathcal{I} = \Phi R_\beta\mathcal{I}$ be a rotation of β and, possibly, a reflection (i.e. $\Phi \in \{\mathrm{Id}, J\}$) applied to $\mathcal{I}$. From the Eq. (9), $\tilde{f}$ reads

$$\tilde{f}(\phi\mathcal{I}) = \frac{1}{2\pi}\int_0^{2\pi}\frac{f(R_\alpha\phi\mathcal{I}) - f(JR_\alpha\phi\mathcal{I})}{2} - \alpha\,d\alpha. \tag{S16}$$

We prove that $\tilde{f}$ respects Eq. (7) by addressing the cases $\Phi = \mathrm{Id}$ (ϕ does not include a reflection) and $\Phi = J$ (ϕ does include a reflection).
Case - $\det(\Phi) = 1$, i.e. $\tilde{f}(\phi\mathcal{I}) = \tilde{f}(R_\beta Id\mathcal{I}) = \tilde{f}(R_\beta\mathcal{I})$.
By definition of $\tilde{f}$, the group average yields

$$\tilde{f}(\phi\mathcal{I}) = \frac{1}{2\pi}\int_0^{2\pi}\frac{f(R_\alpha R_\beta\mathcal{I}) - f(JR_\alpha R_\beta\mathcal{I})}{2} - \alpha\,d\alpha \tag{S17}$$

$$= \frac{1}{2\pi}\int_0^{2\pi}\frac{f(R_{\alpha+\beta}\mathcal{I}) - f(JR_{\alpha+\beta}\mathcal{I})}{2} - \alpha\,d\alpha, \tag{S18}$$

which, after applying the rotation of $\alpha + \beta = \gamma$, becomes

$$= \frac{1}{2\pi}\left(\int_0^{2\pi} \frac{f(R_\gamma \mathcal{I}) - f(JR_\gamma \mathcal{I})}{2} - \gamma d\gamma, + \int_0^{2\pi} \beta\, d\beta\right) \tag{S19}$$

$$= \frac{1}{2\pi}\int_0^{2\pi} \frac{f(R_\gamma \mathcal{I}) - f(JR_\gamma \mathcal{I})}{2} - \gamma\, d\gamma + \beta \tag{S20}$$

$$= \tilde{f}(\mathcal{I}) + \beta \tag{S21}$$

Case 2 - $\det(\Phi) = -1$, i.e. $\tilde{f}(\phi\mathcal{I}) = \tilde{f}(R_\beta J\mathcal{I})$
The group average $\tilde{f}$ yields

$$\tilde{f}(\phi\mathcal{I}) = \frac{1}{2\pi}\int_0^{2\pi} \frac{f(R_\alpha J R_\beta \mathcal{I}) - f(JR_\alpha JR_\beta \mathcal{I})}{2} - \alpha\, d\alpha. \tag{S22}$$

The order in which mirroring and rotation are applied determines the sign of the rotation. For an angle θ, the identity $R_\alpha J\theta = (\pi - \theta) + \alpha = \pi - (\theta - \alpha) = JR_{-\alpha}\theta$ holds. By using this fact, we get

$$= \frac{1}{2\pi}\int_0^{2\pi} \frac{f(R_{-\alpha}R_\beta J\mathcal{I}) - f(JJR_{-\alpha}R_\beta \mathcal{I})}{2} - \alpha\, d\alpha \tag{S23}$$

$$= \frac{1}{2\pi}\int_0^{2\pi} \frac{f(R_{-\alpha+\beta}J\mathcal{I}) - f(R_{-\alpha+\beta}\mathcal{I})}{2} - \alpha\, d\alpha \tag{S24}$$

$$= \frac{1}{2\pi}\int_0^{2\pi} -\frac{f(R_{-\alpha+\beta}\mathcal{I}) - f(JR_{-\alpha+\beta}\mathcal{I})}{2} - \alpha\, d\alpha, \tag{S25}$$

which, after applying the transformation $-\alpha + \beta = \gamma$, becomes

$$= \frac{1}{2\pi}\int_\beta^{-2\pi+\beta} -\frac{f(R_\gamma \mathcal{I}) - f(JR_\gamma \mathcal{I})}{2} + \gamma - \beta(-d\gamma) \tag{S26}$$

$$= \frac{1}{2\pi}\int_0^{-2\pi} \frac{f(R_\gamma \mathcal{I}) - f(JR_\gamma \mathcal{I})}{2} - \gamma + \beta d\gamma \tag{S27}$$

$$= \frac{1}{2\pi}\int_0^{-2\pi} \frac{f(R_\gamma \mathcal{I}) - f(JR_\gamma \mathcal{I})}{2} - \gamma + \beta d\gamma \tag{S28}$$

$$= \frac{-1}{2\pi}\int_0^{2\pi} \frac{f(R_\gamma \mathcal{I}) - f(JR_\gamma \mathcal{I})}{2} - \gamma d\gamma + \beta \tag{S29}$$

$$= -(\tilde{f}(\mathcal{I}) + \beta). \tag{S30}$$

Hence, by combining Eq. (S21) and Eq. (S30), the proposition holds

$$\tilde{f}(\phi\mathcal{I}) = (\tilde{f}(\mathcal{I}) + \beta)\det(\phi) \tag{S31}$$

□

We discretize the otherwise continuous $O(2)$-averaging by considering an equi-spaced sampling of the circle and a random sampling, whose results are reported in Fig. 4.

Time delay of two signals

Let $\theta(\omega)$ and $\theta_v(\omega)$ be the Fourier transform of the signals $\theta(t)$ and $\theta_v(t)$, respectively. By applying the argument operator, $\arg(\cdot)$, we can compute the corresponding phase as function of the frequency, i.e. $\phi_\theta(\omega)$ and $\phi_{\theta_v}(\omega)$. This enables to compute the delay time for each frequency component:

$$\tau(\omega) = \frac{\phi_\theta(\omega) - \phi_{\theta_v}(\omega)}{2\pi}. \tag{S32}$$

We retain as characteristic delay time between the signals the value $\tau(\omega^*)$, provided ω^* exists, according to the following procedure: considering the frequency range $f \in [0.6, 1.2]$ Hz, where the typical walking fluctuations occur, we compute

$$\omega_\theta = \arg\max_{f\in[0.6,1.2]\mathrm{Hz}}(E_\theta) \tag{S33}$$

$$\omega_{\theta_v} = \arg\max_{f\in[0.6,1.2]\mathrm{Hz}}(E_{\theta_v}) \tag{S34}$$

where E_θ and E_{θ_v} are the energy spectra that can be computed by application of the module operator $|\cdot|$ to $\theta(\omega)$ and $\theta_v(\omega)$. We set $\omega^* = \omega_\theta$ if $\omega_\theta \approx \omega_{\theta_v}$, i.e. velocity and orientation are synchronized. Else, we discard the trajectory from the computation of the delay.

Generation of synthetic data

We generate real-life imagelets mimicking the overhead shape of people in terms of a superposition of two ellipses: one for the body/shoulder, E_b, and another one, E_h, at lower depth values (larger height), for the head (cf. Figure 3(a,b)). We characterize each ellipse, by 6 random scalars: Cartesian coordinates x_j, y_j of its center, area A_j, eccentricity e_j, rotation angle α_j and depth value c_j (i.e. the gray shade in the colorization in Fig. 2, $j = b$, h). A bivariate normal random distribution around the imagelet center determines the position of the body ellipse. The head ellipse is superimposed at a uniform random position closely to the end of the body ellipse minor axis. We add exceptions that are often seen in real-life data (i.e. perturbations of the overhead elliptical pedestrian shape) by drawing 4 additional ellipses at random positions in the imagelets. Moreover, artifacts of adjacent pedestrians in imagelets due to local dense situations are represented in the dataset by cropping 9 imagelets from a 3×3 grid in which pedestrians are drawn at random positions relative to these grid points. We represent clothing artifacts, shape variations and imperfect depth reconstruction by applying random modifications to each synthetic imagelet (in the same spirit of [3]): these include pixel removal (i.e. replacing 15% random pixels with background pixel value), pixel addition (i.e. replacing 25% with median pixel value), depth translation (i.e. increasing the values of all foreground pixels with a single uniform random variable $x_i \in [-15, 15]$) and Gaussian noise ($\sigma = 5$). Finally, we smoothen by convolving the imagelets with a 3×3 averaging kernel, resulting in the synthetic imagelets of Figure 3. We report the imagelet generation algorithm in full in Algorithm 1.

Features of the real-life dataset

The real-life dataset that we employ has been collected via a real-life experiment, called "Moving Light" held during week-long 2017 Eindhoven Glow Light Festival (cf. our previous paper on the experiment [4] and festival website [5]). The Glow festival is held in November on a yearly basis. It runs during the evenings and it involves a city-wide uni-directional route in which visitors are walking by exhibits related to illumination design and light art. The Moving Light experiment had the shape of a rectangular corridor exhibit. Visitors walked along a corridor about 24 m long and 6 m wide. In the last 12 m section, visitors were tracked at 30FPS via a home-made high-resolution system based on a grid of 12 Microsoft Kinect™ overhead depth sensors (a sketch of the experimental setup is in Fig. 1 of the manuscript). In the present work, we employed 78k trajectories that have been collected during the first five days of the measurement campaign. During the festival evenings, our installation was crossed by between 50 and 250 pedestrians per minute (see an example evening in Fig. S3(a)). The flow was mostly diluted (average density $\approx 0.37\,\mathrm{ped/m^2}$), with rare density peaks at $2.2\,\mathrm{ped/m^2}$ (see distribution of local density in Fig. S3(b)).

References

[1] Jammalamadaka, S. R. & Sengupta, A. *Topics in circular statistics*, vol. 5 (world scientific, 2001).

[2] MacKay, D. J. C. *Information theory, inference and learning algorithms* (Cambridge university press, 2003).

[3] Corbetta, A., Menkovski, V. & Toschi, F. Weakly supervised training of deep convolutional neural networks for overhead pedestrian localization in depth fields. In *2017 14th IEEE International Conference on Advanced Video and Signal Based Surveillance (AVSS)*, 1–6, DOI: https://doi.org/10.1109/AVSS.2017.8078490 (IEEE, 2017).

[4] Corbetta, A. *et al.* A large-scale real-life crowd steering experiment via arrow-like stimuli. vol. 5, 61–68, DOI: https://doi.org/10.17815/CD.2020.34 (2020).

[5] Authors, V. Glow festival eindhoven. DOI: https://www.gloweindhoven.nl/en (2017).

Algorithm 1 Algorithm for the generation of synthetic imagelets (cf. Fig. 3). Each iteration of the nested for-loop draws a single pedestrian on a square background β at relative distance d to create artifacts of adjacent pedestrians. Random variables (generated in lines $6-19$) characterise two ellipses (drawn in lines 20 and 21) that represent the body and head. We introduce a 25% probability of drawing children, represented by smaller ellipses (lines 18 and 19). Additionally, perturbations of the elliptical overhead shape (e.g. due to backpacks, arms or posture) are imitated by drawing $N_{obj} = 4$ small ellipses at random positions in lines $23-29$. Finally, 9 imagelets are obtained by cropping around each of the 3×3 grid positions (lines 30-36).

INPUT: $N_{obj} = 4, d = 35$
OUTPUT: 9 imagelets $\mathcal{I}$ and 9 labels θ_{gt}

1: $\beta :=$ square background with $(2d+40)^2$ pixels and pixel values 255
2: $\mathcal{I} :=$ object containing imagelets with (40×40) resolution
3: $\theta_{gt} :=$ object containing ground-truth labels

4: **for** $i = 0$ to 3 **do**
5:     **for** $j = 0$ to 3 **do**

6:         $\vec{r}_{grid} := \big((35 + di + \eta_1) \cdot \vec{e}_x, (35 + dj + \eta_2) \cdot \vec{e}_y\big)$ with $\eta_1, \eta_2 \sim \mathcal{N}(0, 2)$
7:         $x_h := r_{grid,x} + \eta$ with $\eta \sim \mathcal{N}(0, 2)$
8:         $y_h := r_{grid,y} + \mu$ with $\mu \sim \mathcal{U}(0, 7)$
9:         $\epsilon_b, \epsilon_h := (\eta_1, \eta_2)$ with $\eta_1, \eta_2 \sim \mathcal{N}(1.6, 0, 2)$
10:         **while** $\epsilon_b < 1.35$ or $\epsilon_h < 1.0$ **do**
11:             $\epsilon_b, \epsilon_h := (\eta_1, \eta_2)$ with $\eta_1, \eta_2 \sim \mathcal{N}(1.6, 0, 2)$
12:         **end while**
13:         $A_b := \mu$ with $\eta \sim \mathcal{N}(600, 80)$
14:         $A_h := A_b \cdot \eta$ with $\eta \sim \mathcal{N}(0.3, 0.03)$
15:         $c_b := \eta$ with $\eta \sim \mathcal{N}(170, 12)$
16:         $c_h := \eta$ with $\eta \sim \mathcal{N}(155, 8)$
17:         $\alpha_b := -\frac{\pi}{2} + \mu$ with $\mu \sim \mathcal{U}(0, \pi)$
18:         $\alpha_h := \alpha_b + \eta$ with $\eta \sim \mathcal{N}\left(-\frac{\pi}{6}, \frac{\pi}{6}\right)$
19:         **while** $\mu > 0.75$ with $\mu \sim \mathcal{U}(0, 1)$ **do**
20:             Reduce A_b, A_h, c_b, c_h with 25%
21:         **end while**

22:         β.DrawEllipse$\big(\vec{r}_{grid}, A_b, \epsilon_b, \alpha_b, c_b,\big)$
23:         β.DrawEllipse $\big((x_h\vec{e}_x, y_h\vec{e}_y), A_h, \epsilon_h, \alpha_h, c_h,\big)$
24:         θ_{gt}.append$\big(\alpha_b\big)$

25:         **for** $k = 0$ to N_{obj} **do**
26:             $\vec{r}_{obj} := \big(\eta \cdot \vec{e}_x, \eta \cdot \vec{e}_y\big)$ with $\eta \sim \mathcal{N}(10, 4)$
27:             $\epsilon_{obj} := \mu$ with $\mu \sim \mathcal{U}(1, 2)$
28:             $A_{obj} := \mu$ with $\eta \sim \mathcal{N}(100, 12)$
29:             $c_{obj} := \eta$ with $\eta \sim \mathcal{N}(170, 10)$
30:             $\alpha_{obj} := -\frac{\pi}{2} + \mu$ with $\mu \sim \mathcal{U}(0, \pi)$
31:             β.DrawEllipse $\big(\vec{r}_{obj}, A_{obj}, \epsilon_{obj}, \alpha_{obj}, c_{obj},\big)$
32:         **end for**

33:     **end for**
34: **end for**
35: **for** $m = 0$ to 3 **do**
36:     **for** $n = 0$ to 3 **do**
37:         $\vec{r}_{crop} := (\big((35 + md) \cdot \vec{e}_x, (35 + nd + \eta) \cdot \vec{e}_y\big)$
38:         img $:= \beta$.Crop$\big($ at $= \vec{r}_{crop}\big)$
39:         img := img.ApplyNoise$\big(\big)$
40:         img := img.Smoothen$\big($kernel $= (3 \times 3)\big)$
41:         $\mathcal{I}$.append$\big($ img $\big)$
42:     **end for**
43: **end for**
return $\mathcal{I}, \theta_{gt}$

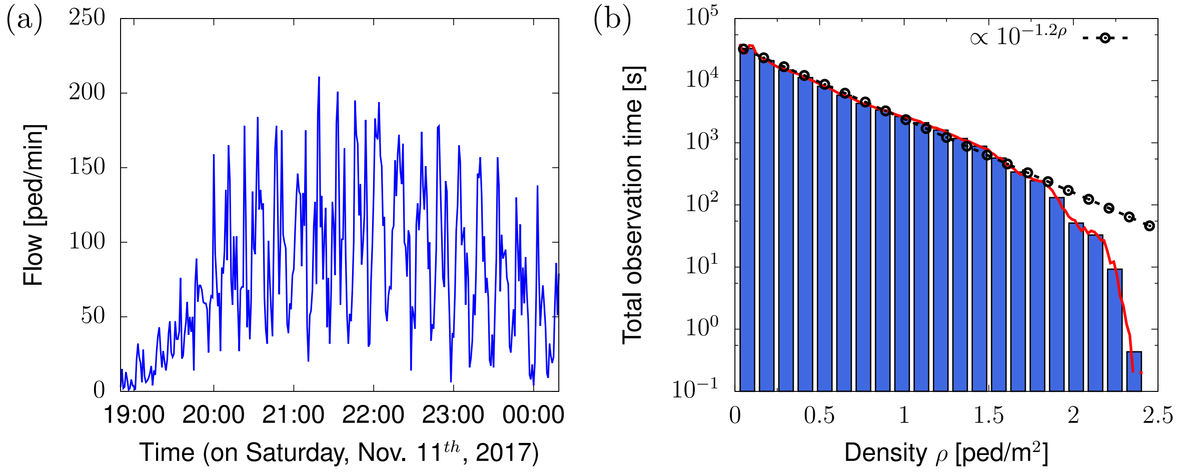

Figure S3: (a) Visitors flow (in pedestrians per minute) for the first evening of the festival (Saturday Nov. 11th 2017). A fluctuation in the number of visitors with ≈ 15 min period can be observed. This was due to a light show occurring four times per hour located few hundred meters upstream our installation. (b) Distribution of observed pedestrian density across all the evenings (in pedestrians per square meter, x-axis, vs. total observation time in seconds, y-axis).
